# Temperature-Dependent Residue Depletion Regularities of Tiamulin in Nile Tilapia (*Oreochromis niloticus*) Following Multiple Oral Administrations

**DOI:** 10.3389/fvets.2021.679657

**Published:** 2021-06-11

**Authors:** Cuiyv Cao, Yongtao Liu, Guodong Zhang, Jing Dong, Ning Xu, Shun Zhou, Yibin Yang, Qiuhong Yang, Xiaohui Ai

**Affiliations:** ^1^Collage of Fisheries and Life Science, Shanghai Ocean University, Shanghai, China; ^2^Yangtze River Fisheries Research Institute, Chinese Academy of Fishery Sciences, Wuhan, China; ^3^Key Laboratory of Control of Quality and Safety for Aquatic Products, Ministry of Agriculture and Rural Affairs, Beijing, China; ^4^Hubei Province Engineering and Technology Research Center for Aquatic Product Quality and Safety, Wuhan, China

**Keywords:** tiamulin, Nile tilapia, tissue residue, temperature, LC-MS/MS

## Abstract

The aim of this study was to investigate the effect of different water temperatures (19, 25, and 30°C) on tissue residue depletion of tiamulin in Nile tilapia (*Oreochromis niloticus*) after five consecutive days of oral administration at the dose of 20 mg/kg body weight and to calculate the corresponding elimination half-life (*T*_1/2_) and withdrawal times (WTs). After oral administration at scheduled 11 time points (1, 2, 3, 5, 7, 9, 12, 15, 20, 25, and 30 days), samples of plasma and tissues (muscle plus skin, liver, kidney, and gill) were collected. Tiamulin concentration in samples were determined by liquid chromatography coupled with tandem mass spectrometry (LC-MS/MS). *T*_1/2_ was calculated by the equation: *T*_1/2_ = ln2/*k*. WT 1.4 software was used to calculate WT. The results showed that tiamulin was widely distributed in all tissue samples with the highest concentration in liver. At three different water temperatures, the *T*_1/2_ were calculated as 2.76, 2.13, and 1.64 days in plasma, 2.71, 1.85, and 1.31 days in muscle plus skin, 2.27, 1.70, and 1.50 days in liver, 2.84, 2.32, and 1.94 day in kidney, and 3.16, 2.42, and 1.74 days in gill, respectively. At 19°C, the order of WT is kidney (11.88 days) > liver (10.41 days) > gill (10.77 days) > plasma (8.83 days) > muscle plus skin (7.14 days). The WT for tiamulin at 25°C was in the following order: kidney (8.40 days) > liver (8.21 days) > gill (8.07 days) > plasma (7.24 days) > muscle plus skin (4.05 days). At 30°C, the WT dropped and shown as follows: gill (6.99 days) > kidney (6.51 days) > liver (6.29 days) > plasma (3.27 days) > muscle plus skin (2.92 days). The present investigations indicated that increasing the temperature from 19 to 30°C shortened *T*_1/2_ and WT of tiamulin in tilapia. To ensure the safety of fish consumption, the longest WT of tissues is suggested for tiamulin in Nile tilapia at the corresponding water temperature; i.e., WTs were 12 days at 19°C, 9 days at 25°C, and 7 days at 30°C, respectively. Overall, we intended to provide a theoretical basis for tissue residue depletion kinetics of tiamulin in fish and improve our understanding of the influence of the temperature on tissue residue depletion kinetics of tiamulin in fish.

## Introduction

Tilapia (*Oreochromis*), accounting for 10% of the world's finfish production, are among the most important aquaculture species of the twenty-first century (1). Nile tilapia (*Oreochromis niloticus*) is the most commonly farmed tilapia species, accounting for 70% of tilapia production (2). In 2017, the output of Nile tilapia was 4,130,281 tons (2), and Nile tilapia has become the third most important fish in aquaculture after carp and salmon (3). However, with the expansion of tilapia culture industry, high-intensity farming and poor water quality are associated with the occurrence of disease (4), which greatly reduced the production of tilapia (5). Previous studies have shown that water temperature appears to be one of the most important factors contributing to the susceptibility of Nile tilapia to pathogenic bacteria infection (6–8). The dynamic distribution and elimination patterns of *Streptococcus agalactiae* in *O. niloticus* were also closely related to water temperature (9). As a consequence, for better prevention and control of disease infection in Nile tilapia, rational use of drugs at different temperature should be taken into account, and it is necessary to investigate the pharmacokinetics and tissue disposition kinetics of drugs in this species at different temperature.

Tiamulin, 14-deoxy-14 [(2-diethylaminoethyl)-mercapto-acetoxy] mutilin, produced by basidiomycete *Pleurotus mutilus*, is a semisynthetic derivative of the diterpene antibiotic pleuromutilin (10). *In vitro* and *in vivo* studies have shown that tiamulin exhibited potent antimicrobic activity against a variety of Gram-positive and Gram-negative bacteria such as *streptococci, mycoplasmas*, as well as *klebsiella* (11, 12). Additionally, tiamulin can also promote the growth of swine and poultry (13), and it has low toxicity and weak accumulation (14). Therefore, this drug has been extensively used for the treatment of bacterial diseases in veterinary medicine. In aquaculture, tiamulin is a very effective bactericidal compound against the majority of *Renibacterium salmoninarum* isolates *in vitro* with a minimum bactericidal concentration (MBC) of 10.95 μg/ml for 90% of the strains tested (15). Investigation of tiamulin against Gram-negative bacterial pathogens of fish was performed *in vitro* and *in vivo*, and the results demonstrated that five strains of *Vibrio anguillarum* were sensitive to tiamulin at 1.6–6.25 μg/ml (16). Enteric redmouth disease induced by *Yersinia rucheri* in rainbow trout can also be controlled by tiamulin (17). Other research also suggested that a small amount of tiamulin could promote the growth of aquatic animals and reduce the harm of diseases (18). For this reason, tiamulin was widely applied for the treatment of various bacterial infections in aquatic animals. On the other hand, because of the extensive use of tiamulin in aquaculture, its potential public health hazard by intake of fish containing tiamulin residue has also received widespread attention. An effective measure to control the drug residues in aquatic animals is setting the rational withdrawal time (WT). Tissue residue depletion study is an important part to determine proper withdrawal periods for drugs used in food fishes (19). However, as far as we know, no information on residue depletion regularities of tiamulin in fish was reported. Moreover, in aquatic animals, the residue depletion and WT of a drug can be deeply affected by environmental factors, such as temperature, pH, and salinity (oxytetracycline in tilapia) of water in which the animals are raised (20, 21). As far as temperature is concerned, because fish are heterothermic animals, a large number of researches showed that the absorption, distribution, biotransformation, and excretion of different drugs in different fish can be seriously affected by temperature, such as florfenicol in Nile tilapia (22, 23), doxycycline in grass carp (24), florfenicol amine in crucian carp (25), and enrofloxacin and its metabolite ciprofloxacin in turbot (26). Therefore, the goal of this study was to explore the residue characterization and acquire the WT of tiamulin in Nile tilapia plasma, muscle plus skin, liver, kidney, and gill at three different water temperatures.

## Materials and Methods

### Chemicals and Reagents

Tiamulin fumarate standard (purity ≥ 98%) was purchased from Dr. Ehrenstorfer (Augsburg, Germany). Tiamulin-^13^C_4_ fumarate (purity ≥ 98%) and internal standard (IS) were supplied by Toronto Research Chemicals (North York, Canada). The tiamulin fumarate technical powder (purity ≥ 98%) used for oral administration was provided by Shandong Lu Kang Animal Medicine Co., Ltd. (Jining, China). LC-MS-grade water was obtained from Merck (Darmstadt, Germany). The HPLC-grade methanol, hexane, acetonitrile, and formic acid were purchased from J.T. Baker (Deventer, Holland). Magnesium sulfate anhydrous was bought from Sinopharm Chemical Reagent Company (Shanghai, China). The anesthetic tricaine methanesulfonate (MS-222) was purchased from Aibo Biotechnology Co. Ltd. (Wuhan, China). Flash C18 powder with a particle size of 40–60 μm was acquired from Agela Technologies (Tianjin, China). Heparin sodium was obtained from Shanghai Biochemical Testing Co. Ltd (Shanghai, China). Centrifugal tubes were provided by Shanghai CNW Technologies (Shanghai, China).

Individual standard stock solutions were prepared by dissolving 10 mg of tiamulin fumarate standard and 10 mg of tiamulin-^13^C_4_ fumarate in acetonitrile and diluted to a final concentration of 100 mg/L, respectively. All standard stock solutions are contained in 100 ml of screw-thread amber glass bottle and stored at −20°C light-free. Then, we prepared the intermediate standard solution of tiamulin fumarate at 10 mg/L by diluting the standard stock solution of tiamulin fumarate at 100 mg/L with the mixture solution of acetonitrile and water (50:50, *v/v*). Intermediate IS solution of tiamulin-^13^C_4_ fumarate at 1 mg/L was made by diluting standard stock solution of tiamulin-^13^C_4_ fumarate at 100 mg/L with the mixture solution of acetonitrile and water (50:50, *v/v*). The solution for oral administration was prepared by dissolving tiamulin fumarate technical powder in distilled water and adjusting to the final concentration of 5 mg/L. Heparin sodium water solution (1%, *w/v*) was prepared by dissolving 1 g of heparin sodium in 100 ml of distilled water.

### Experimental Animals and Experimental Design

All the experimental protocols and procedures involving animals in this study were secured from the Fish Ethics Committee of Yangtze River Fisheries Research Institute, Chinese Academy of Fishery Sciences, Wuhan, China (ID: 2021-Liu Yongtao-01).

Healthy Nile tilapia (mean body weight 100 ± 10 g, mixed genders) used in our study were acquired from the base of the Yangtze River Fisheries Research Institute, Chinese Academy of Fishery Sciences (Jingzhou, China). Then, they were randomly divided into three experimental groups and control groups and reared in aquariums (12 fish each tank provided with 400 L of tap water). The fish were supplied with oxygen by an inflation pump to ensure the dissolved oxygen levels in water were kept close to saturation. The pH value of water was 7.5–8.5. The room temperature was set at 17°C by an air conditioner, and the water temperature was maintained at 19 ± 1°C, 25 ± 1°C, and 30 ± 1°C by means of an aquarium heater, respectively. To avoid differences in drug kinetics being caused by different nutritional status and make the fish adapt to the water temperature, before conducting the experiment, the fish were starved for 5 days. The fish were raised under a photoperiod of 16 h in light and 8 h in darkness throughout the whole experimental period.

### Oral Administration and Sampling

Prior to the administration, the fish were anesthetized with bicarbonate-buffered (1:1) tricaine methanesulfonate (150 μg/L, MS-222) in order to calm the fish for about 30–60 s for drug administration (27, 28). Fish in the experimental group were weighed and administrated with tiamulin solution at a dose of 20 mg/kg body weight by oral gavage directly to the stomach *via* the stomach tube (29). Oral administration was carried out once a day at the same time for 5 days in succession. After everyday oral administration, each fish was observed in a separate tank for a while. If oral solution was regurgitated, the fish was removed from the study and replaced. Then, the fish after treatment were placed in different temperature tanks to recover. The control group was orally administrated with distilled water without medication administration. After the last administration in our study, six fish were taken out from the tanks at each sampling time point including 1, 2, 3, 5, 7, 9, 12, 15, 20, 25, and 30 days. Blood samples were collected from the caudal vessels of each fish (~2 ml/fish) using heparin-coated 2.5-ml syringes and transferred into 10-ml centrifugal tubes with a cap. Then, the blood samples were centrifuged at 4,000 r/min for 5 min at 4°C, and the supernatant plasma was transferred into a new tube. After collecting blood samples, the fish were immediately euthanized by immersion in an overdose of bicarbonate-buffered (1:1) tricaine methanesulfonate (250 mg/L, MS-222) (20) and then the tissues of muscle plus skin, liver, kidney, and gill were also collected from fish. The tissues were homogenized using a homogenizer and separately kept in sealed plastic bags. All samples were immediately transferred to a −80°C refrigerator for storage no more than 1 month until analysis.

### Sample Preparation

The preparation procedure of samples was based on a previous method (30, 31) with some modifications. For plasma sample processing, after the sample was thawed at room temperature, 1 ml of the sample was transferred into 10-ml polypropylene tubes with cap and 50 μl of intermediate IS solution (1 μg/ml) was added to plasma. Afterwards, 5 ml of acetonitrile was added into the sample, and the sample was vortexed for 30 s; then, the sample was placed in an ultrasonic bath for 1 min. Next, 0.4 g of magnesium sulfate anhydrous was added to the sample, which was vortex agitated for 30 s and centrifuged for 5 min at 8,000 r/min. After that, the supernatants were transferred to another tube, the residues were extracted with 3 ml of acetonitrile once again, and the extracts were combined together. The combined extracts were condensed by gentle nitrogen stream at 50°C until the extract solutions were completely dry. The dry residues were dissolved by adding 1 ml of acetonitrile aqueous (50:50, *v/v*), and then 1 ml of hexane was added into the tubes, shaken by a vortex mixer for 30 s, and centrifuged for 5 min at 8,000 r/min. Finally, the lower layer liquid in the tubes was passed through a 0.22-μm nylon syringe filter disc for the analysis.

For the preparation of muscle plus skin, 2 g of homogenized tilapia muscle plus skin was introduced into 15-ml polypropylene tubes, and 100 μl of intermediate IS solution (1 μg/ml) was added into the sample. After that, 7 ml of acetonitrile was added into the sample and shaken for 30 s, followed by ultrasonic treatment for 1 min. One gram of magnesium sulfate anhydrous was added to the sample and the mixture was vortexed for 1 min; then, the sample was centrifuged for 5 min at 8,000 r/min. The supernatant was transferred into another new tube, and the remaining matrix was extracted again by 5 ml of acetonitrile. The resulting upper layer was merged with the former tube, and the extractant was topped up to 12 ml with acetonitrile; 1 g of magnesium sulfate anhydrous and 0.1 g of Flash C18 powder were added, and then the mixture was vortexed for 30 s and centrifuged for 5 min at 8,000 r/min. Next, we accurately removed half of the supernatants into a new tube, and the extracts were condensed by gentle nitrogen stream at 50°C until the extract solutions were completely dry. The dry residues were dissolved by adding 1 ml of acetonitrile aqueous (50:50, *v/v*), and then 1 ml hexane was added to it. The mixture was vigorously shaken for 30 s and centrifuged for 5 min at 8,000 r/min. The lower layer liquid was filtered through 0.22-μm nylon syringe filters for the analysis.

The preparation procedure for the liver (1 g), kidney (1 g), and gill (1 g) was similar to that for muscle plus skin. Tissue samples were subjected to 50 μl of 1 μg/ml intermediate IS solution, and then the extraction and purification protocols were in accordance with the method for muscle plus skin as described above.

### Analytical Method of LC-MS/MS

Tiamulin in plasma and tissues was determined using a high-performance liquid chromatogram tandem mass spectrometry system (Surveyor MS Pump Plus, Surveyor Autosampler Plus and Thermo TSQ Quantum Access MAX, Thermo Scientific, USA). Data were obtained and processed through the Thermo LCquan software (Version 2.6, Thermo Scientific).

Separation of compounds was performed on a 150 × 2.1 mm column packed with 5-μm particles (Hypersil GOLD C18 column, Thermo, USA), and the column temperature was maintained at 35°C. The flow rate was 300 μl/min, and the injection volume was 10 μl in full loop injection mode. Mobile phase consisted of acetonitrile (mobile phase A) and water containing 0.1% formic acid (mobile phase B). The conditions of flow phase gradient elution are as follows: The gradient started with 10% A for 1 min and rose up to 85% A within 4 min, then returned to 10% A in 0.1 min, and finally maintained for 2.9 min. The cycle time was 8.0 min per sample injection.

The MS system was carried out using heated electrospray ionization (HESI) in positive ion mode. We choose the mode of selective reaction monitoring (SRM) to monitor corresponding protonated molecular ion for target analytes. The optimum parameters employed for determination of tiamulin were as follows: vaporizer temperature, 300°C; spray voltage, 3,500 V; sheath gas pressure (high-purity nitrogen), 30 psi; auxiliary gas pressure, 5 arb; ion transport tube temperature, 350°C; collision gas pressure (ultra-high-purity argon), 1.50 mTorr; Q1 peak width of 0.70 amu and Q3 peak width of 0.70 amu; and a scan time of 0.2 s. SRM for fragmentation transitions was *m/z* 494.3–192.0 for tiamulin quantitation with a collision energy of 34 eV and *m/z* 494.3–119.0 for tiamulin quantitation with a collision energy of 21 eV. SRM for tiamulin-^13^C_4_ was *m/z* 498.3–196.0 with a collision energy of 20 eV.

### Validation Procedure

The developed determination method of tiamulin in fish samples was assessed according to the Bioanalytical Method Validation Guidance for Industry (32). The parameters of calibration curves, recovery, precision, matrix effect, limit of detection (LOD), and limit of quantitation (LOQ) were considered to validate the analytical method.

### The Standard Calibration Curves

A solvent standard calibration curve was prepared by plotting the ratios [peak area of tiamulin divided by peak area of 50 μg/L of IS in acetonitrile aqueous (50:50, *v/v*)] vs. tiamulin concentrations of 0, 0.5, 1, 5, 10, 25, 50, 100, 200, 500, and 1,000 μg/L. The matrix-matched calibration curves for tiamulin in various tissues of tilapia were obtained by plotting the ratios of analyte peak area to that of IS (50 μg/L) in blank matrix solution against the concentrations of tiamulin (0, 0.5, 1, 5, 10, 25, 50, 100, 200, 500, and 1,000 μg/L). Blank matrix solutions were made by blank samples referred to the “Sample preparation” method (33). Curve linearity relationship was determined by correlation coefficients (*r*^2^). If the correlation coefficient is at least 0.990 and measured concentrations are within 15% of actual concentrations, the result is acceptable (34). Matrix effects were evaluated by comparing the slopes of the matrix-matched calibration curves vs. solvent standard calibration curves. Generally, it is considered that the matrix effect is not obvious when the ratio is between 85 and 115% (35).

### Recovery, Precision, LOD, and LOQ

Recovery rate was determined by comparing the measured concentration of blank sample spiked before and after preparation. The quality control samples were prepared with spiking a standard solution of tiamulin and IS into blank plasma and tissues of tilapia in the manner of the samples at three concentration levels: 0.5 MRL, MRL, and 1.5 MRL (i.e., 50, 100, and 150 μg/L, MRL = 100 μg/L), and IS at 50 μg/L. Samples were treated as described above, and each concentration was assayed in six replicates. Intra-day and inter-day precision were evaluated *via* calculating the relative error (RE) and relative standard deviation (RSD), which was determined through analyzing six replicates fortified with standard of the analyst at three concentrations (0.5 MRL, MRL, and 1.5 MRL) on the same day and on three different days, respectively. The LOD and the LOQ were defined as the minimum detectable concentration of tiamulin resulting in a peak area of the signal three times and 10-fold than that of the baseline noise in fortified plasma and tissues samples with tiamulin, respectively.

### Data Analysis

The calibration curves were calculated by the computer program Microsoft Office 2019 Excel. The depletion kinetics of tiamulin in fish was fitted to an exponential model by using the following equations: *C*_*t*_ = *C*_0_*e*^−*kt*^, where *C*_*t*_ (μg/kg) represents the tissue concentration of tiamulin at time *t* (h); *C*_0_ (μg/kg) is an extrapolate initial concentration of tiamulin in tissues after 5 days of oral administration; *k* represents the elimination rate constant. The half-life of depletion (*T*_1/2_) was calculated from the equation: *T*_1/2_ = ln2/*k*. According to the tissue drug concentration at different time points to calculate WT. WTs of tiamulin in plasma and other collected tissues were calculated by WT 1.4 software developed by EMA (36).

South Korea's Ministry of Food and Drug Safety established the maximum residual limit (MRL) of tiamulin in fish at 100 μg/kg (37). Therefore, 100 μg/kg was used to calculate the WT of tiamulin in various matrices of Nile tilapia.

## Results

### Method Validation

The solvent standard calibration curve has a good linear correlation over the range of 0–1,000 μg/L with the weighted *r*^2^ of 0.9998. It means that the method had a high selectivity with no interferences. The regression equation is: *y* = 0.0081*x* – 0.0207. LOD was 0.1 μg/L or μg/kg, and LOQ was 0.5 μg/L or μg/kg, respectively, in both plasma and tissues. Recovery rates of tiamulin in plasma and tissues are 96.33–114.06%. Intra-day and inter-day variabilities were 1.26–3.65% and 2.77–5.28%, respectively (Table 1). The results demonstrated that the method had high accuracy and good repetition to quantify tiamulin in plasma and various tissues of tilapia. The equations and correlation coefficients of matrix-matched calibration curves are summarized in Table 2. The results showed that the matrix effect value is between 90.12 and 95.06%. It indicates that the matrix effect is not obvious. To sum up, the method was suitable to be used to quantify tiamulin's concentrations in plasma and various tissues of Nile tilapia.

**Table 1:** Recovery and precision of the method for tiamulin in spiked plasma, muscle plus skin, liver, kidney and gill of Nile tilapia (*n* = 6).

**Tissues**	**Spiked level**	**Recovery (%)**	**Precision (%)**	
		**(μg/kg or μg/L)**		
			**Intra-day**	**Inter-day**
Plasma	50	114.06	3.45	5.28
	100	110.48	3.31	4.28
	150	113.09	2.46	3.67
Muscle plus skin	50	102.07	2.88	4.29
	100	98.20	3.11	5.26
	150	97.23	2.64	4.29
Liver	50	96.33	3.17	4.68
	100	109.73	3.54	5.11
	150	110.64	2.59	5.12
Kidney	50	108.23	3.65	4.18
	100	104.51	3.11	4.25
	150	99.26	2.77	3.55
Gill	50	99.65	2.72	3.68
	100	102.56	2.49	3.89
	150	100.89	1.99	5.01

**Table 2:** The linear range, regression equations, correlation coefficients (*r*^2^) and matrix effects of tiamulin in matrix-matched calibration curves.

**Tissue**	**Linear range**	**Regression equation**	**Correlation coefficients**	**Matrix effect (%)**
	**(μg/kg or μg/L)**		**(*r*^**2**^)**	
Plasma	0–1,000	*y* = 0.0073*x –* 0.0021	0.9991	90.12
Muscle plus skin	0–1,000	*y* = 0.0077*x* + 0.0045	0.9983	95.06
Liver	0–1,000	*y* = 0.0077*x –* 0.0008	0.9972	95.06
Kidney	0–1,000	*y* = 0.0074*x –* 0.0048	0.9979	91.36
Gill	0–1,000	*y* = 0.0087*x* + 0.0033	0.9966	93.10

### Residue Analysis

**Figure 1:**
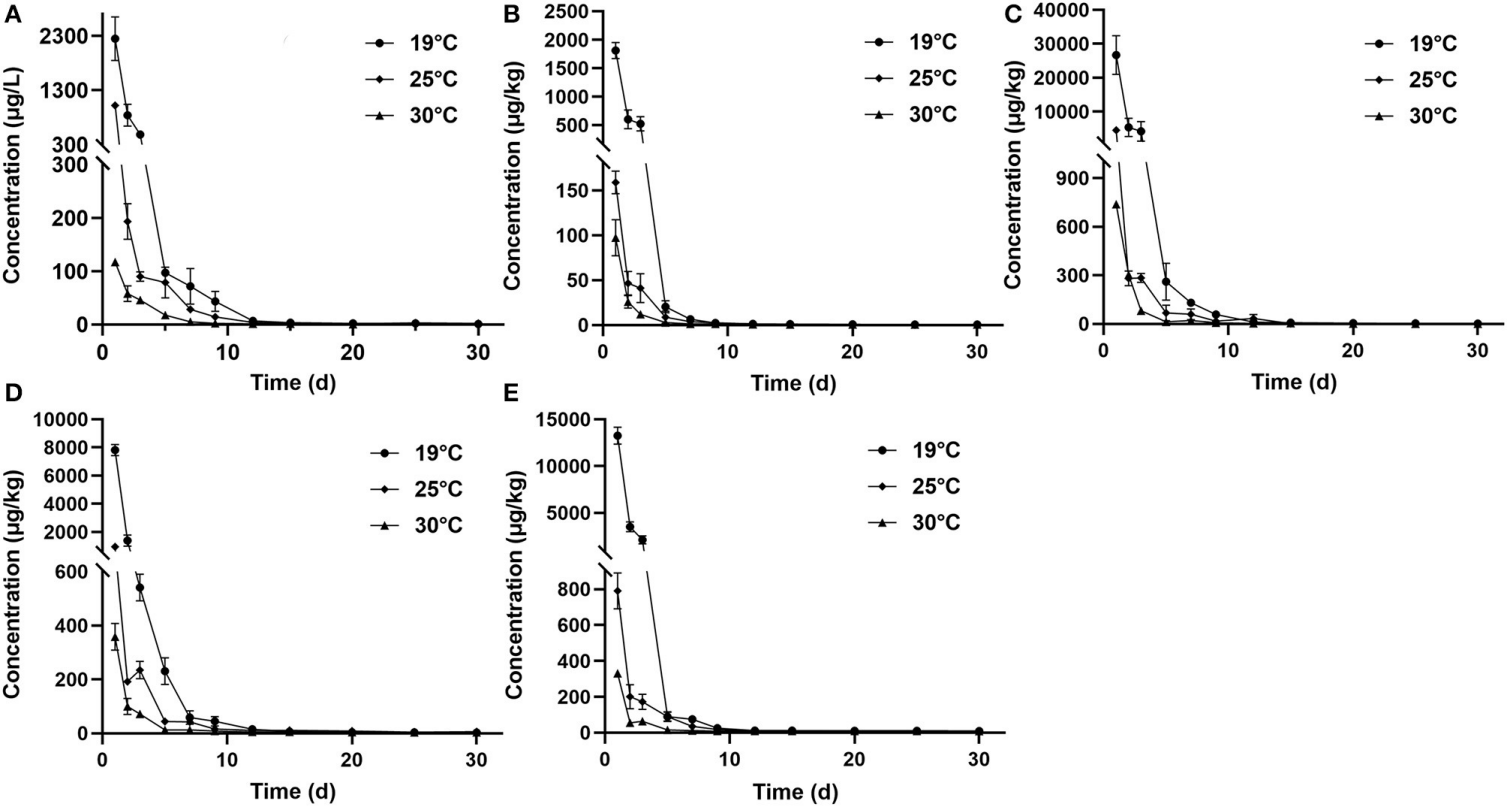
Elimination curve of tiamulin in plasma **(A)**, muscle plus skin **(B)**, liver **(C)**, kidney **(D)**, and gill **(E)** of Nile tilapia after continuous oral administration of 20 mg/kg body weight for 5 days at three temperatures.

The concentration data of tiamulin in plasma and tissues of Nile tilapia after oral dosing at 20 mg/kg at three different temperatures are shown in Tables 3A–C. The residue depletion kinetic curves are shown in Figure 1. According to the results, at three water temperatures, tiamulin were detected in all tissues. When water temperature is 19°C, the result showed that the highest level of tiamulin in plasma and tissues (muscle plus skin, liver, kidney, and gill) was observed on day 1 after administration. Tiamulin concentration in plasma and tissues of Nile tilapia was in the following order: liver > gill > kidney > plasma > muscle plus skin, and the corresponding highest mean concentration of tiamulin was 26,675.64, 13,263.26, 7823.07, 2247.34, and 1813.40 μg/kg or μg/L, respectively. Afterwards, the concentrations of tiamulin in liver, gill, kidney, plasma, and muscle plus skin decreased to 260.36, 89.05, 230.26, 97.31, and 20.41 μg/kg or μg/L on the 5th day, respectively. Except for the liver and kidney, the level of tiamulin in other tissues was below the MRL set by South Korea (100 μg/kg). When water temperature is 25°C, according to the data, the maximum level of tiamulin was also determined on the first day after oral administraiton; the order of initial concentration is liver > plasma > kidney > gill > muscle plus skin, and the highest average concentration is 4509.53, 1018.59, 935.29, 790.39, and 158.93 μg/kg or μg/L, respectively. At 25°C, the highest concentration in plasma is about 45% compared to that at 19°C, 9% in muscle plus skin, 17% in liver, 12% in kidney, and 6% in gill. The concentration at the 5th day after the last medication is well below the MRL (100 μg/kg). When water temperature is 30°C, the order of initial residual level is liver > kidney > gill > plasma > muscle plus skin, and the highest average concentration is 738.50, 357.55, 330.26, 117.25, and 97.22 μg/kg or μg/L, respectively. At the second time point (2nd day after medication), tiamulin levels in plasma, muscle plus skin, kidney, and gill were below 100 μg/kg, and on the 3rd day, the level of tiamulin in liver was below 100 μg/kg. Compared to the highest level at 19 and 30°C, the values at 30°C were decreased by 95% in plasma, muscle plus skin, and kidney, and 97% in liver and gill.

**Table 3A:** The tiamulin concentrations in plasma and tissues of Nile tilapia after daily oral administration of a single dose of 20 mg/kg for 5 days at 19°C (*n* = 6).

**Time**	**Plasma**	**Muscle plus skin**	**Liver**	**Kidney**	**Gill**
**(day)**	**(μg/L)**	**(μg/kg)**	**(μg/kg)**	**(μg/kg)**	**(μg/kg)**
1	2247.34 ± 550.08	1813.40 ± 140.69	26675.64 ± 5727.44	7823.07 ± 159.31	13263.26 ± 898.29
2	840.33 ± 390.15	603.23 ± 162.22	5327.68 ± 2732.93	1386.45 ± 184.12	3528.77 ± 535.39
3	483.60 ± 202.84	525.37 ± 123.85	4200.06 ± 2898.73	540.09 ± 395.61	2127.45 ± 438.60
5	97.31 ± 7.29	20.41 ± 6.73	260.36 ± 114.62	230.26 ± 93.04	89.05 ± 27.80
7	71.68 ± 33.48	6.48 ± 0.35	131.47 ± 22.84	58.96 ± 25.01	74.40 ± 0.00
9	43.38 ± 18.66	2.44 ± 0.43	58.34 ± 8.02	44.48 ± 17.91	25.93 ± 2.03
12	6.98 ± 4.81	1.42 ± 0.33	11.03 ± 4.05	15.38 ± 6.65	11.98 ± 1.47
15	3.36 ± 0.76	1.05 ± 0.11	8.13 ± 4.53	5.80 ± 0.02	12.01 ± 1.04
20	2.23 ± 0.14	0.88 ± 0.13	5.04 ± 3.30	3.40 ± 1.22	10.72 ± 1.96
25	2.27 ± 0.80	0.69 ± 0.11	3.60 ± 2.58	3.66 ± 0.54	10.25 ± 0.09
30	1.18 ± 0.08	0.62 ± 0.19	1.70 ± 0.69	3.27 ± 1.29	9.24 ± 0.15

**Table 3B:** The tiamulin concentrations in plasma and tissues of Nile tilapia after daily oral administration of a single dose of 20 mg/kg for 5 days at 25°C (*n* = 6).

**Time**	**Plasma**	**Muscle plus skin**	**Liver**	**Kidney**	**Gill**
**(day)**	**(μg/L)**	**(μg/kg)**	**(μg/kg)**	**(μg/kg)**	**(μg/kg)**
1	1018.59 ± 36.99	158.93 ± 12.56	4509.53 ± 159.76	935.29 ± 31.41	790.39 ± 258.38
2	193.26 ± 33.26	46.69 ± 13.14	281.00 ± 45.97	191.65 ± 3.97	200.39 ± 66.84
3	89.93 ± 9.00	41.15 ± 16.18	283.63 ± 28.42	234.60 ± 32.00	172.31 ± 41.57
5	78.92 ± 28.91	8.66 ± 6.87	67.22 ± 49.07	43.59 ± 12.51	90.24 ± 25.93
7	28.48 ± 5.25	3.94 ± 1.30	59.29 ± 33.11	42.46 ± 12.52	35.15 ± 11.00
9	14.63 ± 2.72	2.54 ± 0.18	16.86 ± 11.84	15.65 ± 1.07	17.83 ± 3.35
12	4.10 ± 0.56	1.36 ± 0.42	33.74 ± 24.87	11.24 ± 4.50	12.16 ± 1.10
15	2.16 ± 0.42	0.63 ± 0.09	2.41 ± 0.23	10.88 ± 3.82	12.29 ± 0.42
20	1.48 ± 0.00	0.55 ± 0.01	3.05 ± 0.77	8.33 ± 1.83	10.64 ± 0.92
25	2.92 ± 0.92	0.42 ± 0.03	1.70 ± 0.58	4.22 ± 0.21	10.30 ± 0.34
30	2.10 ± 0.66	0.30 ± 0.03	1.02 ± 0.53	6.05 ± 2.56	9.29 ± 0.35

**Table 3C:** The tiamulin concentrations in plasma and tissues of Nile tilapia after daily oral administration of a single dose of 20 mg/kg for 5 days at 30°C (*n* = 6).

**Time**	**Plasma**	**Muscle plus skin**	**Liver**	**Kidney**	**Gill**
**(day)**	**(μg/L)**	**(μg/kg)**	**(μg/kg)**	**(μg/kg)**	**(μg/kg)**
1	117.25 ± 5.32	97.22 ± 20.03	738.50 ± 12.61	357.55 ± 58.91	330.26 ± 21.59
2	57.90 ± 14.60	25.73 ± 7.04	303.95 ± 5.60	99.53 ± 29.83	55.23 ± 8.91
3	45.99 ± 4.83	12.00 ± 1.41	81.00 ± 23.54	71.65 ± 1.60	64.00 ± 7.33
5	17.63 ± 7.20	2.79 ± 0.29	13.23 ± 2.65	12.80 ± 6.14	15.86 ± 2.37
7	5.28 ± 1.71	1.28 ± 0.14	21.38 ± 8.75	12.38 ± 7.28	11.54 ± 1.22
9	2.44 ± 0.86	1.42 ± 0.39	7.07 ± 2.41	8.27 ± 2.53	9.54 ± 0.44
12	1.34 ± 0.62	0.93 ± 0.19	3.50 ± 1.60	5.30 ± 1.49	9.30 ± 0.26
15	1.26 ± 0.37	0.58 ± 0.07	5.05 ± 1.55	5.53 ± 1.63	8.77 ± 0.26
20	0.55 ± 0.01	0.70 ± 0.18	1.67 ± 0.55	4.54 ± 0.65	8.85 ± 0.88
25	0.33 ± 0.02	0.54 ± 0.12	1.19 ± 0.15	3.49 ± 0.50	8.71 ± 0.16
30	0.15 ± 0.01	0.70 ± 0.41	0.81 ± 0.09	3.95 ± 1.01	7.82 ± 0.13

In the same tissues, there was a negative correlation between the water temperature and the maximum concentration of tiamulin. Tiamulin concentration at the first sampling time point of 1 day after the last oral administration was the highest value and then began to decrease. For example, in liver, on day 1, the levels of tiamulin at 19, 25, and 30°C are 26,675.64 μg/kg, 4509.53 μg/kg, and 738.50 μg/kg, respectively; on day 5, the levels of tiamulin at the three temperatures are 260.36, 67.22, and 13.23 μg/kg, respectively. Compared with the other tissues, the levels of tiamulin were highest in liver while muscle plus skin was lowest at the three water temperatures.

The depletion equation, correlation coefficient (*r*^2^), and elimination half-life (*T*_1/2_) of tiamulin in tilapia are listed in Tables 4A–C. At three different water temperatures, the *T*_1/2_ was calculated as 2.76, 2.13, and 1.64 days in plasma, 2.71, 1.85, and 1.31 days in muscle plus skin, 2.27, 1.70, and 1.50 days in liver, 2.84, 2.32, and 1.94 days in kidney, and 3.16, 2.42, and 1.74 days in gill, respectively.

**Table 4A:** The equation of elimination curve, correlation coefficient (*r*^2^) and elimination half-life (*T*_1/2_) of Nile tilapia after daily oral administration of a single dose of 20 mg/kg for 5 days at 19°C.

**Tissue**	**Equation of elimination curve**	**Correlation coefficient (*r*^**2**^)**	**Elimination half-life (*T*_**1/2**_)/d**
Plasma	*C_*Plasma*_* = 611.2*e^−^*^0.251t^	*r*^2^ = 0.8489	2.76
Muscle plus skin	*C_*Muscle*_* = 202.76*e^−^*^0.256t^	*r*^2^ = 0.6792	2.71
Liver	*C_*Liver*_* = 3225*e^−^*^0.305t^	*r*^2^ = 0.8032	2.27
Kidney	*C_*Kidney*_* = 958.59*e^−^*^0.244t^	*r*^2^ = 0.7874	2.84
Gill	*C_*Gill*_* = 1143.8*e^−^*^0.219t^	*r*^2^ = 0.6258	3.16

**Table 4B:** The equation of elimination curve, correlation coefficient (*r*^2^) and elimination half-life (*T*_1/2_) of Nile tilapia after daily oral administration of a single dose of 20 mg/kg for 5 days at 25°C.

**Tissue**	**Equation of elimination curve**	**Correlation coefficient (*r*^**2**^)**	**Elimination half-life (*T*_**1/2**_)/d**
Plasma	*C_*Plasma*_* = 390.64*e*^−0.325**t**^	*r*^2^ = 0.9075	2.13
Muscle plus skin	*C_*Muscle*_* = 103.77*e*^−0.374**t**^	*r*^2^ = 0.9283	1.85
Liver	*C_*Liver*_* = 1272.2*e*^−0.407**t**^	*r*^2^ = 0.821	1.70
Kidney	*C_*Kidney*_* = 444.36*e*^−0.299**t**^	*r*^2^ = 0.8378	2.32
Gill	*C_*Gill*_* = 429.1*e*^−0.286**t**^	*r*^2^ = 0.8741	2.42

**Table 4C:** The equation of elimination curve, correlation coefficient (*r*^2^) and elimination half-life (*T*_1/2_) of Nile tilapia after daily oral administration of a single dose of 20 mg/kg for 5 days at 30°C.

**Tissue**	**Equation of elimination curve**	**Correlation coefficient (*r*^**2**^)**	**Elimination half-life (*T*_**1/2**_)/d**
Plasma	*C_*Plasma*_* = 145.27*e*^−0.423**t**^	*r*^2^ = 0.9761	1.64
Muscle plus skin	*C_*Muscle*_* = 79.235*e*^−0.53**t**^	*r*^2^ = 0.8784	1.31
Liver	*C_*Liver*_*= 507.05*e*^−0.462**t**^	*r*^2^ = 0.8648	1.50
Kidney	*C_*Kidney*_* = 212.98*e*^−0.357**t**^	*r*^2^ = 0.842	1.94
Gill	*C_*Gill*_* = 213.82*e*^−0.398**t**^	*r*^2^ = 0.8232	1.74

**Figure 2:**
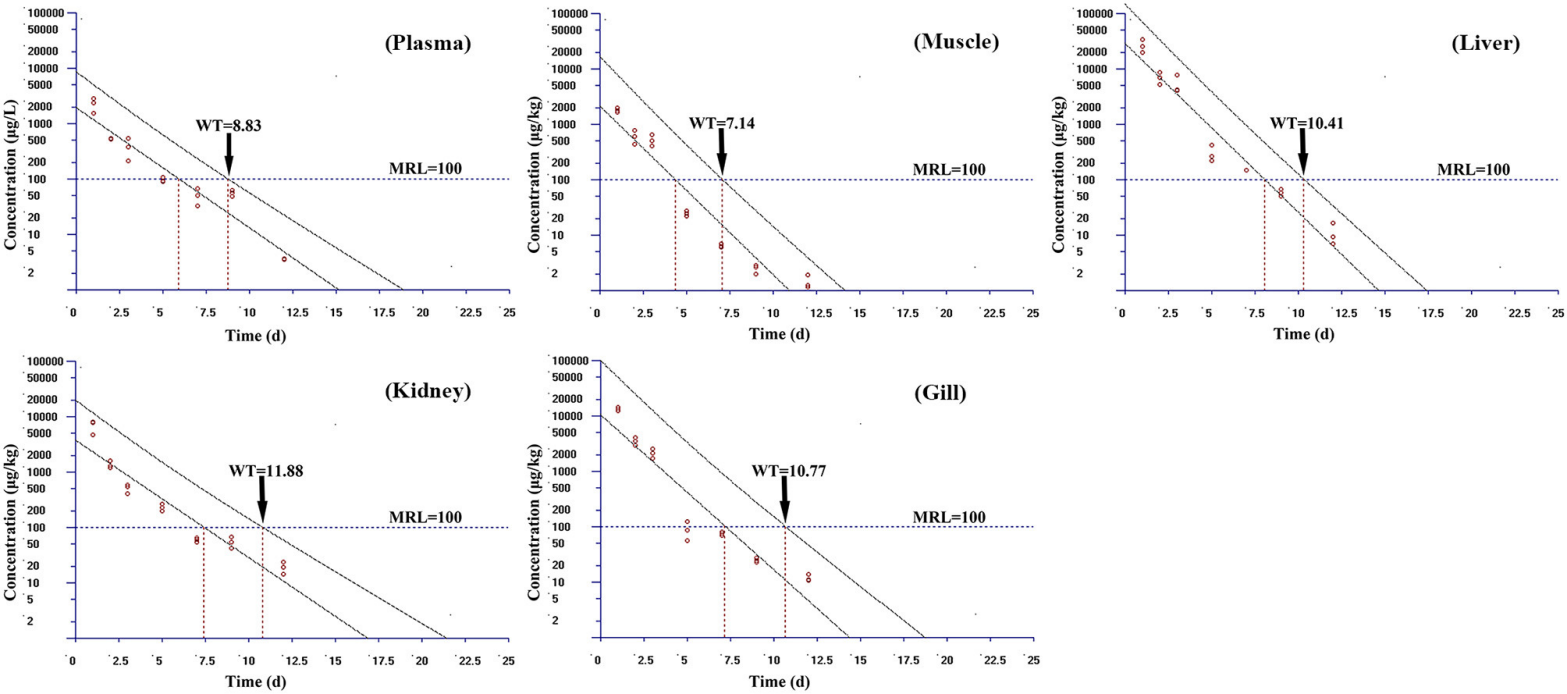
The withdrawal time (WT) of tiamulin in plasma, muscle plus skin, liver, kidney, and gill at 19°C calculated by WT 1.4 software.

**Figure 3:**
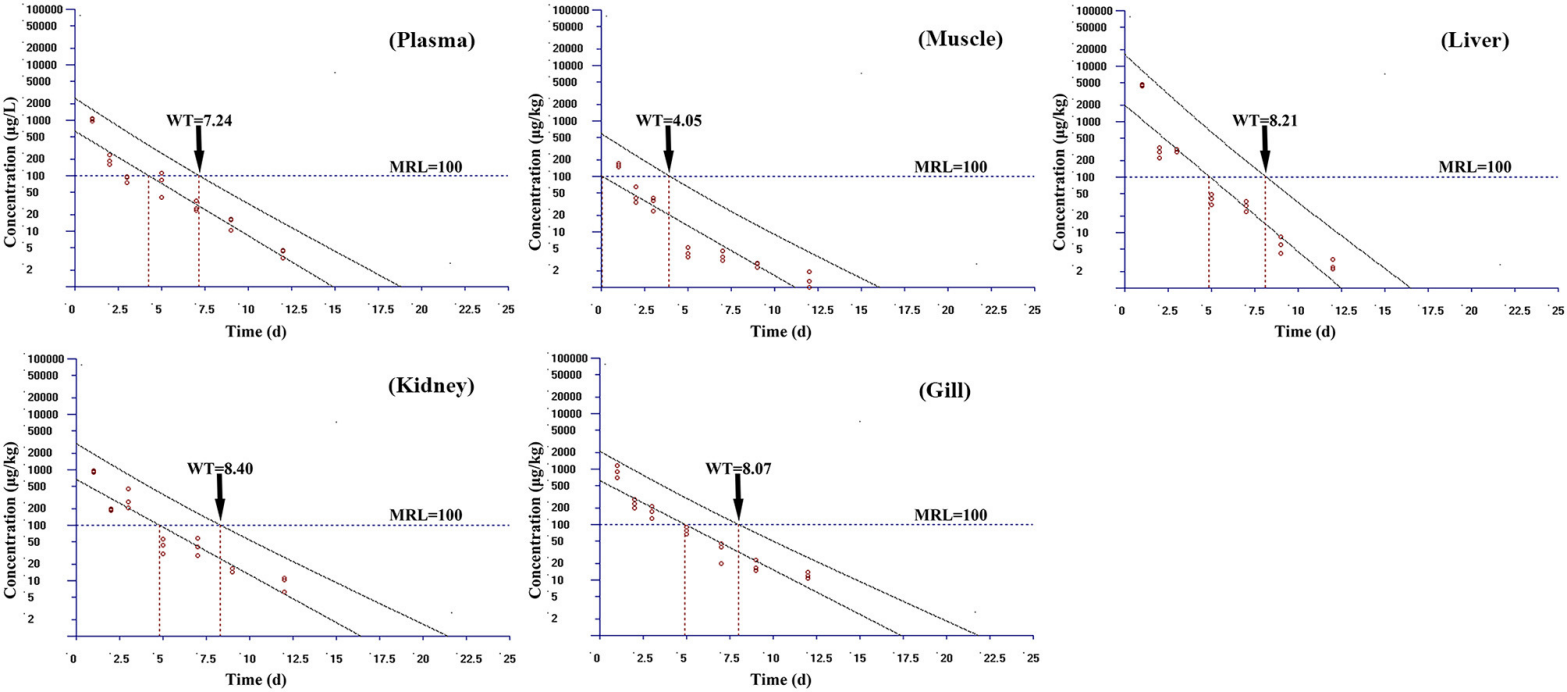
The withdrawal time (WT) of tiamulin in plasma, muscle plus skin, liver, kidney, and gill at 25°C calculated by WT 1.4 software.

**Figure 4:**
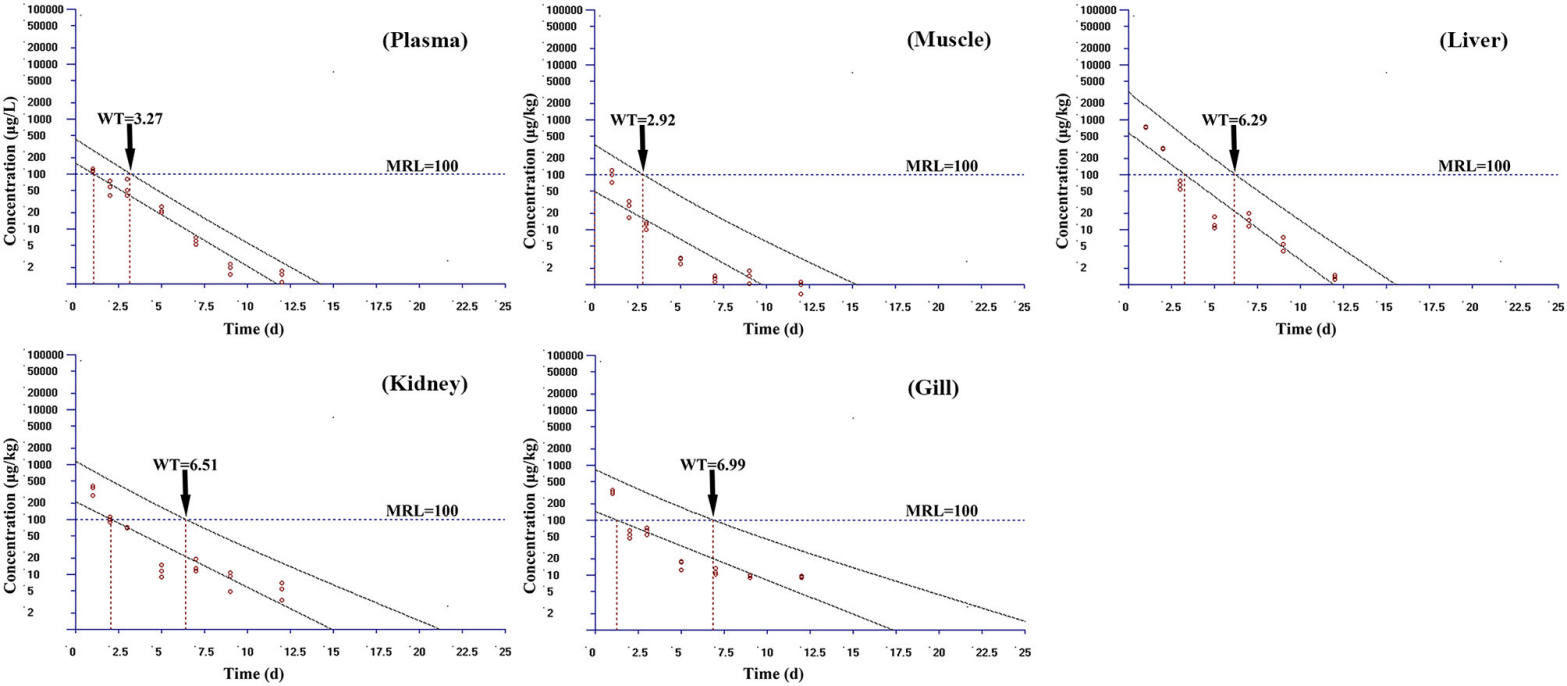
The withdrawal time (WT) of tiamulin in plasma, muscle plus skin, liver, kidney, and gill at 30°C calculated by WT 1.4 software.

Based on the MRL (100 μg/kg) of tiamulin in fish laid down by South Korea, the WT 1.4 software (developed by EMA) was used to estimate the WTs of tiamulin considering a 95% confidence level (36), and the WTs are presented in Figures 2–4. At 19°C, the order of WT is kidney (11.88 days) > liver (10.41 days) > gill (10.77 days) > plasma (8.83 days) > muscle plus skin (7.14 days). The WT for tiamulin at 25°C was in the following order: kidney (8.40 days) > liver (8.21 days) > gill (8.07 days) > plasma (7.24 days) > muscle plus skin (4.05 days). At 30°C, the WTs dropped and are as follows: gill (6.99 days) > kidney (6.51 days) > liver (6.29 days) > plasma (3.27 days) > muscle plus skin (2.92 days). Obviously, as temperature increases, the WT becomes short at the same tissues. The value of WT also demonstrated that kidney, liver, and gill were the major metabolism organs of tiamulin in tilapia, and edible fish tissues (muscle plus skin) have the shortest WT. To ensure the safety of fish consumption, the longest WT is suggested for tiamulin in Nile tilapia at the corresponding water temperature, i.e., WTs were 12 days at 19°C, 9 days at 25°C, and 7 days at 30°C, respectively.

## Discussion

This is the first investigation to explore the residue depletion regularities of tiamulin in fish. The findings of this study revealed that tiamulin, upon oral administration to Nile tilapia, is widely distributed in all tissues sampled with the highest concentrations in liver. This finding is similar to earlier studies in broiler chicken following drinking water or feed at a dose of 40 mg/kg body weight for 3 days (38) and in ducks following oral administration at 40 mg/kg/day for 3 days at 23–27°C (39). It has been reported that tiamulin has a high lipid solubility (40), which results in extensive distribution throughout the tissues. Except for the liver, our results showed that gill and kidney had higher concentrations of tiamulin than plasma and muscle plus skin at three water temperatures, because the kidney is a major metabolism and elimination organ and gill plays a significant role in absorption and excretion of drug in fish (41).

Based on the MRL of tiamulin (100 μg/kg) in fish laid down by South Korea (37), the calculated WT values of tiamulin were 8.40 days for kidney, 8.21 days for liver, 8.07 days for gill, 7.24 days for plasma, and 4.05 days for muscle plus skin at 25°C. The results of our study are consistent with the previous results that tiamulin in poultry and swine has a short withdrawal period (38, 39, 42). For example, the calculated WT was 6 days for liver and 5 days for muscle in ducks following oral administration *via* gavage with tiamulin at 40 mg/kg/day for 3 days at 23–27°C (39). Vinothini et al. (38) reported that tiamulin had no residues on day 5 in broiler chicken following treatment with water or feed containing tiamulin at a dose of 40 mg/kg body weight for 3 days, and its WT was <5 days. Crivineanu et al. (42) also estimated the WT of 6 days in pork and 5 days in liver for tiamulin in pigs after receiving drinking water of tiamulin in a dose of 12 mg/kg/day for 5 days. Compared to the reported WT values of tiamulin in poultry or swine, the calculated WT value for tiamulin in liver of fish is distinctly longer than poultry and swine. On the contrary, the value of WT for tiamulin in muscle plus skin of fish in this study is a little shorter than that in poultry and swine. The difference of WT in liver may be because fish belong to poikilotherms having lower activity of drug metabolic enzymes than in poultry and swine.

In this study, the temperature-dependent residue depletion regularities of tiamulin in Nile tilapia were examined for the first time. The results showed that the longest WT of tiamulin in tilapia is 12 days at 19°C, 9 days at 25°C, and 7 days at 30°C, respectively, which suggested that the WT with water temperature exhibited negative correlation within a certain temperature range. Similar results were also reported in other drug studies at different water temperatures. For instance, the WT of enrofloxacin was dropped from 180 to 150 days in channel catfish (*Ietalurus punetaus*) after gavage administration at 20 mg/kg/day for 7 days from 18 to 28°C (43). The WT of doxycycline was also reduced from 55 to 30 days in channel catfish following oral administrations at a dose of 20 mg/kg for five consecutive days from 18 to 28°C (44). These differences in WT are described above in relation to animal species, water temperature, and type of drugs.

Moreover, the water temperature has a remarkable effect on elimination half-life (*T*_1__/2_). At three different water temperatures, *T*_1/2_ values were calculated as 2.76, 2.13, and 1.64 days in plasma, 2.71, 1.85, and 1.31 days in muscle plus skin, 2.27, 1.70, and 1.50 days in liver, 2.84, 2.32, and 1.94 days in kidney, and 3.16, 2.42, and 1.74 days in gill, respectively. It indicated that the *T*_1/2_ decreased from a low temperature to a high temperature of tiamulin in Nile tilapia. Other temperature-dependent studies of drugs in fish also showed a similar tendency. For example, the *T*_1__/2_ of florfenicol in Nile tilapia declined from 12–13 h to 7–8 h at 32°C compared to 24°C at a dose of 15 mg/kg (22). Xu et al. (24) evaluated the effect of temperature on doxycycline in grass carp (*Ctenopharyngodon idella*) after a single oral administration at 20 mg/kg; the results indicated that with the increase of temperature from 18 to 24°C, the tissue *T*_1__/2_ decreased from 27.80 to 17.93, from 51.58 to 37.56, from 66.17 to 23.68, and from 30.08 to 24.22 h for liver, kidney, gill, and muscle plus skin, respectively. The *T*_1__/2_ of florfenicol in crucian carp (*Carassius auratus*) was reduced from 31.79 to 18.33, from 45.29 to 6.26, from 16.15 to 12.44, and from 35.54 to 10.34 day for skin, liver, kidney, and muscle, respectively (25). Other temperature-dependent studies also showed a similar tendency (45, 46).

The results showed that the water temperature had a direct effect on the absorption and distribution of the drug. High water temperature is conducive to the rapid elimination of tiamulin from tissues. It has been proven long time ago that the body temperature of a poikilotherm is generally the same as the surrounding water or slightly higher than the environment (47, 48). The effect of increasing temperature on WT and *T*_1/2_ values has been, in part, because the rise in temperature promotes the change in the physiology conditions of fish, such as shortening blood circulation time, enhancing cardiac output, and increasing metabolic rates of organs (49, 50). Therefore, water temperature is an important factor that should be considered during administration, especially when the water temperature is low, and the withdrawal period should be extended appropriately. In addition, note that the present study used oral administration at 20 mg/kg for 5 days with samples collected up to 30 days after drug treatment. The present study design is suitable to calculate elimination half-life and the withdrawal period in fish at different temperatures, but not sufficient to characterize the pharmacokinetics of tiamulin in fish. Therefore, future studies that determine the pharmacokinetics of tiamulin in fish after a single oral exposure of tiamulin with collected samples in 7 days are necessary in order to understand the dynamic change of tiamulin in fish.

## Conclusion

As far as we know, temperature-dependent residue depletion regularities of tiamulin in aquatic animals were first exhibited in our investigation. We found that tiamulin is widely distributed in all tissues sampled with the highest concentration of tiamulin detected in liver compared to other tissues. What is more, WTs of tiamulin in Nile tilapia after five daily oral administrations at 20 mg/kg are 12 days for 19°C, 9 days for 25°C, and 7 days for 30°C. The present study improves our understanding of the tissue residue depletion kinetics of tiamulin after repeated oral exposure in fish, and the results provide insight into the regulatory decision on the proper withdrawal period of tiamulin in fish under different temperatures to ensure fish-derived food safety.

## Data Availability Statement

The original contributions presented in the study are included in the article/supplementary material, further inquiries can be directed to the corresponding author.

## Ethics Statement

All the experimental protocols and procedures involving animals in this study were secured from the Fish Ethics Committee of Yangtze River Fisheries Research Institute, Chinese Academy of Fishery Sciences, Wuhan, China (ID: 2021-Liu Yongtao-01).

## Author Contributions

CC and YL conceived and designed the animal study and drafted the manuscript. YL and XA provided the facilities to conduct the study and coordinated the project. GZ, YY, and SZ were responsible for rearing fish and collecting samples. JD, QY, and NX determined target compounds in samples. All authors have read and approved the final manuscript.

## Conflict of Interest

The authors declare that the research was conducted in the absence of any commercial or financial relationships that could be construed as a potential conflict of interest.
